# Lipidized Prolactin-Releasing Peptide as a New Potential Tool to Treat Obesity and Type 2 Diabetes Mellitus: Preclinical Studies in Rodent Models

**DOI:** 10.3389/fphar.2021.779962

**Published:** 2021-11-18

**Authors:** Lucia Mráziková, Barbora Neprašová, Anna Mengr, Andrea Popelová, Veronika Strnadová, Lucie Holá, Blanka Železná, Jaroslav Kuneš, Lenka Maletínská

**Affiliations:** ^1^ Institute of Organic Chemistry and Biochemistry, Czech Academy of Sciences, Prague, Czech; ^2^ Institute of Physiology, Czech Academy of Sciences, Prague, Czech

**Keywords:** prolactin-releasing peptide, rodent models, obesity, type 2 diabetes, leptin resistance

## Abstract

Obesity and type 2 diabetes mellitus (T2DM) are preconditions for the development of metabolic syndrome, which is reaching pandemic levels worldwide, but there are still only a few anti-obesity drugs available. One of the promising tools for the treatment of obesity and related metabolic complications is anorexigenic peptides, such as prolactin-releasing peptide (PrRP). PrRP is a centrally acting neuropeptide involved in food intake and body weight (BW) regulation. In its natural form, it has limitations for peripheral administration; thus, we designed analogs of PrRP lipidized at the N-terminal region that showed high binding affinities, increased stability and central anorexigenic effects after peripheral administration. In this review, we summarize the preclinical results of our chronic studies on the pharmacological role of the two most potent palmitoylated PrRP31 analogs in various mouse and rat models of obesity, glucose intolerance, and insulin resistance. We used mice and rats with diet-induced obesity fed a high-fat diet, which is considered to simulate the most common form of human obesity, or rodent models with leptin deficiency or disrupted leptin signaling in which long-term food intake regulation by leptin is distorted. The rodent models described in this review are models of metabolic syndrome with different severities, such as obesity or morbid obesity, prediabetes or diabetes and hypertension. We found that the effects of palmitoylated PrRP31 on food intake and BW but not on glucose intolerance require intact leptin signaling. Thus, palmitoylated PrRP31 analogs have potential as therapeutics for obesity and related metabolic complications.

## Introduction

Obesity, along with type 2 diabetes mellitus (T2DM), is reaching pandemic levels worldwide, and both are a prerequisite for the development of metabolic syndrome (MetS), which culminates in an increased risk of metabolic and cardiovascular diseases (Said, Mukherjee, and Whayne 2016; Engin 2017; Tune et al., 2017). Although it is clear that obesity is linked to an unhealthy lifestyle in today’s society and that adjusting eating habits and lifestyle can partially address obesity issues, new pharmacological treatments are urgently required. Unfortunately, despite the huge efforts to find weight-lowering pharmacotherapies, only a few anti-obesity drugs have recently become available (Rodgers, Tschöp, and Wilding 2012; Kumar 2019; Rose, Bloom, and Tan 2019; Williams, Nawaz, and Evans 2020). One of the promising tools for the treatment of obesity and other related metabolic complications is anorexigenic peptides that are synthetized endogenously in the brain or in the gastrointestinal tract and act centrally to decrease energy intake. As demonstrated in experimental models, these peptides have minimal side effects during long-term anti-obesity treatment (Arch 2015; Patel 2015; Bray et al., 2016).

In their natural form, anorexigenic peptides have several disadvantages for direct use in pharmacotherapy for obesity, mainly due to their chemical instability, short half-life and low brain penetrance through the blood–brain barrier (BBB) after peripheral application. A peptidomimetic approach to modify natural peptides is currently being used for the development of promising drugs (Kumar 2019). The problem of penetration through the BBB can be solved, for example, by coupling of peptides to fatty acids, e.g., palmitic acid, resulting in increased stability and half-life in organisms (Malavolta and Cabral 2011; Salameh and Banks 2014).

Some lipidized peptide-based drugs for treatment of diabetes or obesity have been introduced into the market, such as the insulin analog detemir, which employs myristic acid attached through an amide bound to insulin molecules (Havelund et al., 2004), and liraglutide, a palmitoylated agonist of glucagon-like peptide 1 (GLP-1) (Gault et al., 2011). In addition, liraglutide has been approved for anti-obesity treatment in the United States and Europe (Saxenda). Very recently, a once-weekly injection of the lipidized GLP-1 agonist semaglutide was approved by the FDA for treatment of obesity (Wegovy). Several other peptidomimetics, including multitargeted molecules—dual and triple agonists targeting GLP-1, glucagon and gastric inhibitory polypeptide receptors—are in clinical trials as possible future anti-obesity drugs (Williams, Nawaz, and Evans 2020).

Several neuropeptides of brain origin have been demonstrated to have an anorexigenic effect in animal models, such as prolactin-releasing peptide (PrRP), cocaine- and amphetamine-regulated transcript (CART) peptide, α-melanocyte-stimulating hormone (α-MSH) and melanin-concentrating hormone (MCH) (Kunes et al., 2016; Mikulaskova et al., 2016). A lipidized α-MSH analog has been shown to be stable and exert a strong anorexigenic effect (Fosgerau et al., 2014); however, further research was terminated because of its adverse effects on the skin (Royalty et al., 2014).

Prolactin-releasing peptide was initially isolated from the hypothalamus as a ligand for the human orphan G-protein coupled receptor (GPR10) (Hinuma et al., 1998) as a possible regulator of prolactin secretion from anterior pituitary cells. However, later findings suggested that prolactin release is likely not a primary function of PrRP (Jarry et al., 2000; Taylor and Samson 2001). Shortly after its discovery, it was established that PrRP has other physiological functions, particularly it has been found to be involved in food intake, body weight (BW) and energy expenditure regulation (Lawrence et al., 2000; Takayanagi et al., 2008; Atanes, Ashik, and Persaud 2021). There are two biologically active isoforms of PrRP, with either 20 (PrRP20) or 31 (PrRP31) amino acids. Both isoforms have a common C-terminal Arg-Phe-amide sequence, which is critical for their biological activity (Roland et al., 1999; Maletínská et al., 2011). The fact that PrRP is involved in food intake and BW regulation is supported by the detection of PrRP and its receptor GPR10 in brain areas involved in food intake regulation, such as several hypothalamic nuclei (e.g., nucleus arcuatus (Arc), paraventricular nucleus (PVN), dorsomedial nucleus (DMN)) and the brainstem (e.g., nucleus tractus solitarius (NTS)). PrRP was also found to have high affinity for the neuropeptide FF2 (NPFF2) receptor, resulting in anorexigenic effects (Engström et al., 2003). It has also been shown in rodents that intracerebroventricular injection of natural PrRP20 and PrRP31 decreased food intake and BW (Lawrence et al., 2000; Ellacott et al., 2003; Maixnerová et al., 2011). Coadministration of PrRP and adipose tissue-born long-term acting regulator of energy balance leptin in rats resulted in additive reductions in nocturnal food intake and BW gain and an increase in energy expenditure (Ellacott et al., 2002).

Furthermore, both GPR10 knockout mice and PrRP-deficient mice developed late-onset obesity and exhibited a significant decrease in energy expenditure compared to wild-type mice (Bjursell et al., 2007) as well as altered insulin sensitivity and lipid homeostasis (Prazienkova et al., 2021). Moreover, PrRP-deficient mice also displayed increased food intake and attenuated responses to food intake, lowering the cholecystokinin (CCK) and leptin signals (Takayanagi et al., 2008). Therefore, PrRP or its receptor(s) might be new targets in obesity treatment.

However, as a centrally released and centrally acting neuropeptide, natural PrRP has several limitations after peripheral administration: low stability in the organism to exert its central effect and inability to reach the target brain receptors. To overcome these disadvantages, we designed analogs of PrRP lipidized at the N-terminal region, which is not essential for biological activity (Maletínská et al., 2015; Kunes et al., 2016). Our earlier studies demonstrated that analogs lipidized by 8–18 carbon chain fatty acids at the N-terminus of PrRP20 or PrRP31 showed high binding affinities with a K_i_ in the nanomolar range for both GPR10 and the NPFF2 receptor, similar to analogs that were palmitoylated through linkers to Lys^11^ (e.g., palm^11^-PrRP31) (Maletínská et al., 2015; Pražienková et al., 2017; Karnošová et al., 2021).

It was confirmed that lipidization increased the stability of these peptides, as palmitoylated PrRP31 (palm-PrRP31) and myristoylated PrRP20 (myr-PrRP31) were stable for more than 24 h in rat plasma (Zemenová et al., 2017). *In vivo* pharmacokinetics studies in mice also showed longer stability for lipidized analogs than for natural, nonlipidized PrRP31 (Maletínská et al., 2015). The long-lasting anorexigenic effect of lipidized analogs of PrRP could be explained by their prolonged stability owing to binding to serum albumin, similar to liraglutide, semaglutide or palmitoylated gastric inhibitory polypeptide (Gault et al., 2011; Lau et al., 2015; Bech et al., 2017).

Acute *in vivo* experiments demonstrated that lipidized PrRP analogs have central anorexigenic effects after peripheral administration. Our work further supports several indirect studies confirming that the food intake-lowering effect of these analogs is mainly central. There was a significant and dose-dependent decrease in food intake in lean overnight-fasted or freely fed mice after subcutaneous (SC) injection of palm-PrRP31, myr-PrRP20 (Maletínská et al., 2015) or palm^11^-PrRP31 (Pražienková et al., 2017; Pirnik et al., 2021), while analogs lipidized with shorter carbon chains or natural PrRP20 or PrRP31 had no effect on food intake (Maletínská et al., 2015). Moreover, neuronal activity (manifested by increased expression of the immediate early gene c-Fos in brain areas related to food intake regulation) was significantly increased in specific brain nuclei or in areas such as the Arc, PVN, DMN and NTS 90 min after SC application of myr-PrRP20, palm-PrRP31 and palm^11^-PrRP31 but not after natural PrRP31 or octanoyl-PrRP31 administration (Maletínská et al., 2015; Pražienková et al., 2017; Pirník et al., 2018). The central neuronal activation of c-Fos after peripheral application of palmitoylated PrRP is also supported by the selective activation of specific hypothalamic oxytocin and hypocretin neuronal subpopulations both involved in food intake regulation (Pirnik et al., 2015). Furthermore, double c-Fos-GPR10 immunostaining in the brainstem C1/A1 cell group indicated that neurons containing GPR10 receptors are activated after administration of palmitoylated PrRP (Mikulášková et al., 2016).

In the hypothalamus, leptin receptor and PrRP are colocalized and have additive anorexigenic effects. Intracerebroventricular coadministration of PrRP and leptin in rats resulted in additive decrease in food intake and BW loss and an increase in energy expenditure (Ellacott et al., 2002). Furthermore, PrRP-expressing neurons in brain regions involved in food intake regulation (ventromedial nucleus of hypothalamus and ventrolateral medulla and NTS of brainstem) also contain leptin receptors (Ellacott et al., 2002). An anorexigenic effect of PrRP independent of leptin but dependent on the peripheral short-term anorexigenic hormone CCK was suggested in the brainstem. CCK was shown to have no effect on food intake in GPR10-knockout mice. This finding suggests that PrRP acting through its receptor may be a key mediator in the central satiating action of CCK (Bechtold and Luckman 2006).

An exogenously influenced CCK system was also shown to be involved in the central anorexigenic effect of peripherally applied palm-PrRP (Pirnik et al., 2021). We can thus hypothesize that peripheral signals (leptin, CCK) and the central neuropeptide PrRP cooperate in the stimulation of food intake-regulating pathways, leading to a decrease in food intake.

In this review, we summarize the preclinical results of our chronic studies on the pharmacological role of the two most potent palmitoylated PrRP31 analogs with the following sequences: palm-PrRP31 (N-palm)SRAHQHSNleETRTPDINPAWYTGRGIRPVGRF-NH_2_) and palm^11^-PrRP31 (SRTHRHSMEIK(N-γ-E (N-palm))TPDINPAWYASRGIRPVGRF-NH_2_).

These analogs were tested in various mouse and rat models of obesity, glucose intolerance/insulin resistance and T2DM resulting from high-fat (HF) diet feeding (diet-induced obesity (DIO) models) or in rodents with nonfunctional leptin signaling due to a spontaneous mutation in the leptin receptor.

Each of these rodent models represents different types and severities of pathological features of MetS, i.e., 1/obesity as shown by increased BW, triacylglycerides, free fatty acids, cholesterol and/or liver steatosis, 2/prediabetes or T2DM as shown by increased glucose and insulin levels and glucose intolerance, 3/leptin and/or insulin resistance as shown by disrupted peripheral and central leptin or insulin signaling and 4/hypertension as shown by increased blood pressure. All pathologies were compared with that of age-matched control rodents. Chronic peripheral interventions with both palmitoylated PrRP31 analogs in different models allowed us to describe different metabolic changes in these models and to clarify the interactions with other systems involved in food intake regulation, such as the leptin system.

## Chronic Treatment With Palmitoylated PrRP31 Analogs in Mouse and Rat Models of Metabolic Diseases

One of the major risks for the development of cardiovascular and metabolic dysfunction, including obesity, prediabetes and hypertension, is high dietary fat intake. Hypercaloric diets rich in lipids are widely used in experimental studies to induce metabolic disorders commonly found in humans (Dourmashkin et al., 2005; Buettner, Schölmerich, and Bollheimer 2007; Agahi and Murphy 2014). Most rodents tend to become obese and develop pathologies of MetS when fed specific calorie-rich diets (Shafrir, Ziv, and Mosthaf 1999; Bergman et al., 2006; Varga et al., 2010). Frequently used models are mice or rats fed a HF diet.

On the other hand, genetic factors undoubtedly play an important role in obesity development, and it is important to better understand the role of specific factors in food intake regulation using models with genetically disrupted production or signaling of these factors. One of the most important hormones regulating long-term energy balance in organisms is leptin, and the most widely used rodent models of spontaneous genetic obesity and related complications are congenital leptin- or leptin receptor-deficient mice and rats (Varga et al., 2010; Wang, Chandrasekera, and Pippin 2014; Fuchs et al., 2018).

In our studies summarized in this review, various mouse and rat models with different features of MetS were used to investigate the effects of palmitoylated PrRP analogs as potential anti-obesity and antidiabetic compounds and to explore their mechanism of action. Each of these models show a variety of pathologies, and the basic characterization of each model is shown in Table 1.

**TABLE 1:** Characterization of rodent models used in studies of interventions with palmitoylated PrRP31 analogs.

Model	Characterization	References
DIO mice C57BL/6J	Obesity, prediabetes, disturbed central leptin and insulin signaling, liver steatosis	(Maletínská et al., 2015; Pražienková et al., 2017; Holubová et al., 2018)
DIO rats	Obesity, diabetes, glucose intolerance	Holubova et al. (2016); Čermáková et al. (2019)
Sprague-Dawley and Wistar Kyoto		
Ob/ob mice	Severe early onset obesity, disrupted production of leptin, severe liver steatosis, glucose intolerance, disturbed central leptin and insulin signaling	Korinkova et al. (2020)
MSG mice	Obesity, glucose intolerance, hormone disbalance, disrupted hypothalamic leptin and insulin signaling	Špolcová et al. (2015)
ZDF rats	Lean, severe T2DM	Holubova et al. (2016)
Koletsky rats	Obesity, prediabetes, hypertension, liver steatosis, disrupted central leptin and insulin signaling	Mikulaskova et al. (2018)

### DIO Models

DIO rodents are considered models of the most common type of human obesity, which is associated with overconsumption of HF food (Bagnol et al., 2012). To test the effect of chronic treatment with palmitoylated PrRP31 analogs on obesity and prediabetes parameters and on temporarily disturbed central leptin and insulin signaling, we used several mouse and rat models in our studies.

C57BL/6 mice fed a HF diet containing 60% fat based on lard from 8 to 19 weeks of age developed severe obesity and prediabetes (Pelantová et al., 2016). Consumption of the HF diet resulted in significant BW gain in the mice, mediated by an increase in body fat and liver weight and an increased level of leptin, as shown in Table 2. HF diet feeding induced an increase in the mRNA expression of genes involved in lipogenesis in adipose tissue but did not affect the mRNA expression of genes involved in lipolysis. The HF diet also increased the blood glucose level and the insulin and triacylglycerides (TAG) levels in plasma compared to mice on a standard chow diet (LF—low fat diet) (Pelantová et al., 2016).

**TABLE 2:** Summary of metabolic and morphometric parameters in DIO models and impact of treatment with palm-PrRP31 or palm^11^-PrRP31.

Model	Characterization/treatment	BW change	Cumulative food intake	Liver weight	Glucose	Insulin	Leptin	TAG	CHOL	FFA
DIO C57	HF vs LF	↑ 63%	NT	NT	↑	↑	↑	↑	NT	NS
DIO Sprague Dawley	HF vs LF	↑ 22%	↓	NS	NS	↑	↑	NS	NT	↑
DIO Wistar Kyoto	HF vs LF	↑ 10%	NT	NS	↑	NS	NS	NS	NS	NS
DIO C57	palm-PrRP31	↓13%	↓	NS	NS	↓	↓	NT	NT	NT
	palm^11^-PrRP31	↓12%	NS	↓	NS	↓	↓	↓	↓	↓
	palm^11^-PrRP31	↓13, 6%	↓	NS	NS	NS	↓	NS	↓	NS
DIO Sprague Dawley	palm-PrRP31	↓8%	↓	NS	↑	NS	NS	NS	NS	NS
DIO Wistar Kyoto	palm^11^-PrRP31	↓7, 7%	NT	NS	NS	NS	NS	NS	NS	NS

Statistical analysis was performed by unpaired *t*-test, significance is shown as increased (↑) or decreased (↓) vs LF or treatment vs HF saline treated group. Cummulative food intake and body weight (BW) change measured at the end of experiment. Cholesterol (CHOL), free fatty acid (FFA) and triacylglycerides (TAG) measured from the plasma. Non-significant (NS), not-tested (NT) (Maletínská et al., 2015; Holubova et al., 2016; Pražienková et al., 2017; Holubová et al., 2018; Mikulaskova et al., 2018; Čermáková et al., 2019; Korinkova et al., 2020).

In the studies of Maletínská (Maletínská et al., 2015) and Pražienkova (Pražienková et al., 2017), C57BL/6 male mice were provided with a HF diet from 8 to 19 weeks of age to induce obesity. Subsequently, mice were treated SC with saline or palmitoylated analogs of PrRP, palm-PrRP31 or palm^11^-PrRP31 twice a day for 2 weeks. Palm-PrRP31 treatment significantly decreased cumulative food intake. Both palm-PrRP31 and palm^11^-PrRP31 significantly decreased BW, which was primarily mediated by a reduction in body fat and liver, accompanied by a decrease in leptin levels (Table 2).

Due to the decrease in mRNA expression of fatty acid synthase (*Fasn*) in both adipose tissue and the liver along with decreased expression of acetyl-CoA carboxylase (*Acaca*) and sterol regulatory element-binding protein (*Srebp*) in the liver, BW reduction most likely resulted from decreased *de novo* lipogenesis, owing primarily to negative energy balance due to reduced food intake (Maletínská et al., 2015; Pražienková et al., 2017). Moreover, increased uncoupling protein 1 (*UCP-1*) mRNA in brown adipose tissue (BAT) after palm^11^-PrRP31 treatment points to a possible increase in energy expenditure. Furthermore, treatment with both palm-PrRP31 and palm^11^-PrRP31 significantly lowered insulin levels in the blood of DIO mice, and the levels of free fatty acids (FFAs), cholesterol (CHOL) and TAG were significantly reduced after palm^11^-PrRP31 treatment (Table 2).

**FIGURE 1:**
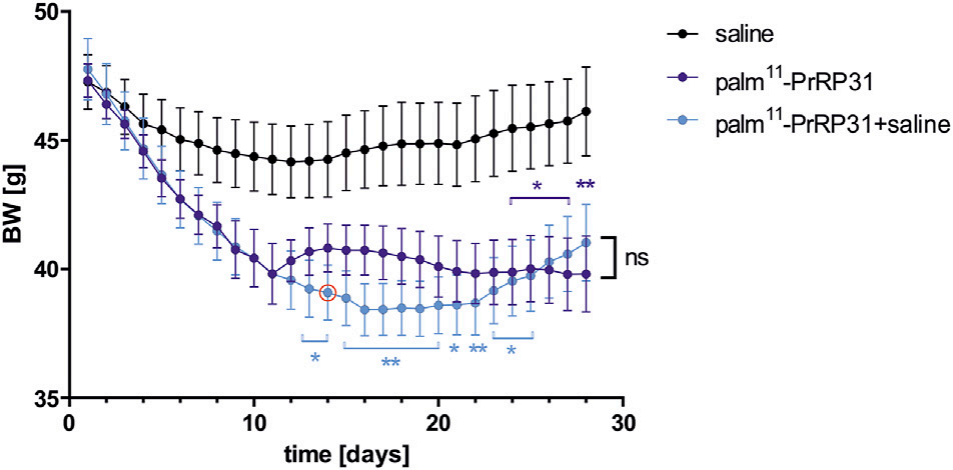
Effect of chronic treatment with palm^11^-PrRP31 on BW in DIO mice. Mice were treated with palm^11^-PrRP31 for 4 weeks (palm^11^-PrRP31 group) or with palm^11^-PrRP31 for 2 weeks and for the following 2 weeks with saline (palm^11^-PrRP31 + saline group), change of the treatment marked by red circle , modified from (Holubová et al., 2018). Data are presented as means ± S.E.M. Statistical analysis was performed by Two-way ANOVA with Tukey *post hoc* test, significance is *p*<0.05, **<0.01 vs saline treated group. Body weight (BW) measured during the treatment, diet-induced obesity (DIO).

The next study of Holubová (Holubová et al., 2018) aimed at palm^11^-PrRP31 posttreatment regarding a possible yo-yo effect after drug termination. C57BL/6 mice were fed for 12 weeks with a HF diet. At the age of 19 weeks, mice were SC injected twice a day with saline for 4 weeks, with palm^11^-PrRP31 for 4 weeks or with palm^11^-PrRP31 for 2 weeks and with saline for the following 2 weeks. DIO mice treated for 4 weeks with palm^11^-PrRP31 and those treated with palm^11^-PrRP31 for 2 weeks and then with saline for 2 weeks reached a similar decrease in BW and body fat and attenuated plasma leptin, which continued for 2 weeks after termination of the 2 weeks-long administration of palm^11^-PrRP31 (Figure 1).

mRNA expression of lipolytic enzymes was significantly lowered by the action of palm^11^-PrRP31 in the liver, suggesting complexly attenuated liver lipid metabolism. Furthermore, similar to our previous study, UCP-1 in BAT points to increased energy expenditure. Under both treatment modes, neuronal activity was increased in food intake-regulating neurons, as determined by FosB expression, a marker of long-term neuronal potentiation (Nestler 2001). Blood glucose, insulin, TAG, FFA and CHOL in plasma were not significantly affected by any of the treatments.

Furthermore, in this study, palm^11^-PrRP31 impacted hypothalamic signaling by restoring the leptin receptor-induced phosphatidylinositol-3-kinase (PI3K) pathway and increasing extracellular signal regulated kinase (ERK) 1/2 phosphorylation as a result of increased leptin or PrRP receptor signaling (Balland and Cowley 2015). Moreover, in this study, palm^11^-PrRP31 lowered the phosphorylation of both c-Jun and c-Jun N-terminal kinases (JNKs), generally activated by HF feeding in DIO mice, both in the periphery and the brain (De Souza et al., 2005; Holubová et al., 2018).

Collectively, studies in DIO mouse models revealed a long-lasting effect of palmitoylated analogs of PrRP31 on BW lowering, accompanied by increased neuronal signaling in the hypothalamus, even after discontinuation of treatment.

In the following studies, we aimed to examine the effects of intraperitoneal (IP) administration of palmitoylated analogs of PrRP31 in rats fed a HF diet that developed not only severe obesity and prediabetes but also glucose intolerance. Sprague–Dawley rats were provided a HF diet from 8 to 32 weeks and subsequently treated with either saline or palm-PrRP31 for 2 weeks (Holubova et al., 2016). Wistar Kyoto rats were fed a HF diet from 8 to 23 weeks of age. At the age of 23 weeks, the mice were IP injected for 3 weeks with either saline or palm^11^-PrRP31 (Čermáková et al., 2019).

The HF diet resulted in significant BW gain, mediated by an increase in body fat and liver weight and an increased level of leptin, as shown in Table 2 (Holubova et al., 2016; Čermáková et al., 2019). Furthermore, the consumption of the HF diet significantly increased intolerance to glucose, determined by an oral glucose tolerance test (OGTT) in both rat strains, with significantly increased fasting blood glucose in Sprague–Dawley rats (Holubova et al., 2016) and increased insulin levels in Wistar Kyoto rats compared to those in the low-fat (LF) diet-fed group.

**FIGURE 2:**
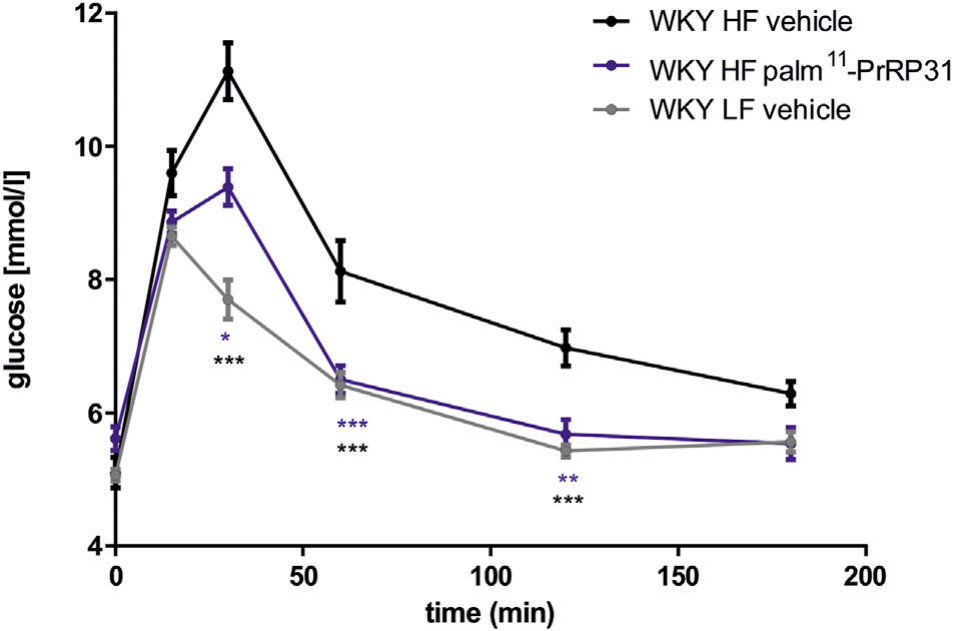
Effect of chronic treatment with palm^11^-PrRP31 on glucose tolerance response in DIO Wistar Kyoto (WKY) rats. Rats were treated with palm^11^-PrRP31 for 3 weeks (WKY HF palm^11^-PrRP31 group). Oral glucose tolerance test (OGTT) was performed after overnight fasting at the end of experiment. Results are shown as a glucose profile modified from (Čermáková et al., 2019). Data are presented as means ± S.E.M. Statistical analysis was performed by Two-way ANOVA with Tukey *post hoc* test, significance is *p*<0.05, **<0.01, ***<0.001 vs WKY HF vehicle treated group. DIO Diet induced obesity, HF high fat, LF low fat.

Similar to mice with DIO, treatment with palm-PrRP31 significantly decreased cumulative food intake, corresponding to a significant decrease in BW in DIO rats after treatment with both palmitoylated analogs (Table 2), primarily mediated by a reduction in body fat and liver weight. In these studies, a significant glucose-lowering effect of both PrRP31 analogs was found in DIO rats after the OGTT but not in the saline-treated control group (Figure 2). Treatment with PrRP31 analogs significantly decreased expression of the enzymes that catalyze *de novo* lipogenesis in both the liver (Holubova et al., 2016; Čermáková et al., 2019) and adipose tissue (Čermáková et al., 2019), while the mRNA expression of lipolytic enzymes was increased after palm^11^-PrRP31 treatment, supporting previous results of complexly affected lipid metabolism. Furthermore, the expression of insulin receptor substrate (Irs) 1 and Irs-2 was increased after palm^11^-PrRP31 treatment. Insulin, TAG, FFA and CHOL in plasma were not significantly affected by any treatment.

In conclusion, in DIO rat models, both palmitoylated analogs of PrRP31 exhibited not only a strong effect on BW lowering but also a great glucose-lowering effect.

### Rodent Models With Leptin Deficiency or Disrupted Leptin Signaling

To test the chronic effect of palmitoylated PrRP31 analogs on obesity and prediabetes or diabetes parameters in relation to the important long-term food intake regulator leptin, we used several mouse and rat models with spontaneous leptin deficiency or disrupted leptin signaling.

Leptin-deficient o*b/ob* mice were used to explore the potential interaction between leptin and PrRP with regard to their anorexigenic effect and impact on metabolic disturbances (Korinkova et al., 2020). In this study, younger mice (treated from 8 to 10 weeks of age) and older mice (treated from 16 to 24 weeks of age) were used. Younger mice were used because they are in a metabolically active state, and older mice have established morbid obesity.

**FIGURE 3:**
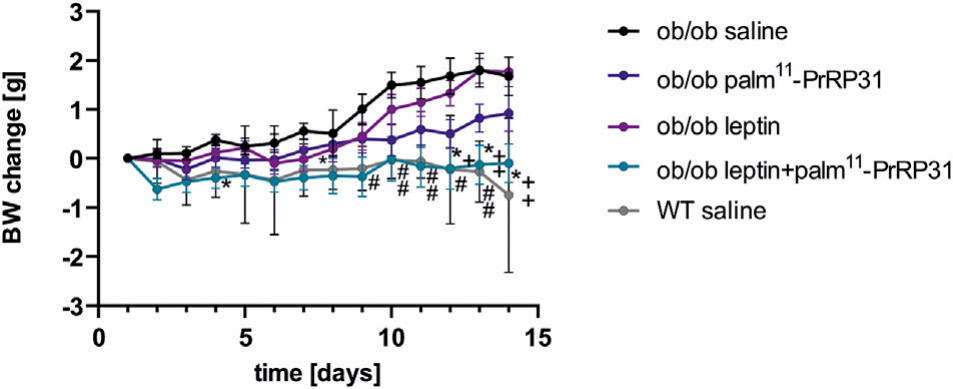
Chronic effect of palm^11^-PrRP31, leptin and their combination on BW change in *ob/ob* mice modified from (Korinkova et al., 2020) Mice were treated fo 2 weeks. Data are presented as means ± S.E.M. Statistical analysis was performed by Two-way ANOVA with Tukey *post hoc* test, significance is ^#^<0.05, ^##^<0.01 *ob/ob* saline vs wild type (WT) saline, *p*<0.05, **<0.01 *ob/ob* leptin + palm^11^-PrRP31 vs *ob/ob* saline, ^+^<0.05, ^++^<0.01 *ob/ob* leptin + palm^11^-PrRP31 vs *ob/ob* leptin. Body weight (BW) measured during the treatment, wild-type.

*Ob/ob* mice of both ages had significantly higher BW, body fat and liver weight than wild-type (WT) mice. As *ob/ob* mice are known to be hypothermic (Ohtake, Bray, and Azukizawa 1977), their rectal temperature was significantly lower at both 10 and 24 weeks of age. Older *ob/ob* mice had high hyperinsulinemia and significantly increased cholesterol levels, but TAG and FFA levels did not differ from those of WT mice (Table 3). Our results supported the study of Enser (Enser 1972), who found that *ob/ob* mice are hyperglycemic only between 5 and 16 weeks of age; however, 24 week-old *ob/ob* mice were normoglycemic in our study (Korinkova et al., 2020). It was demonstrated that nonfunctional leptin receptor signaling leads to negligible PrRP mRNA expression (Ellacott et al., 2002), suggesting interaction of both systems. In this study, neither palm^11^-PrRP31 nor leptin alone significantly decreased the BW, body fat or liver weight of *ob/ob* mice, but their combination significantly lowered all these parameters (Figure 3). Moreover, an increase in the rectal temperature in older *ob/ob* mice was detected after treatment with a combination of leptin and palm^11^-PrRP. Reduced liver weight in younger *ob/ob* mice treated with a leptin and palm^11^-PrRP31 combination was linked to decreased mRNA expression of lipogenic enzymes in the liver and with regression of fat droplets in liver tissue in all groups of younger peptide-treated *ob/ob* mice compared to *ob/ob* saline-treated mice (Korinkova et al., 2020). Treatment with leptin and the combination of palm^11^-PrRP31 + leptin also had a significant decreasing effect on cumulative food intake and total plasma cholesterol levels. In the hypothalamus of older *ob/ob* mice, two main leptin anorexigenic signaling pathways, namely, Janus kinase (JNK)/signal transducer and activator of transcription-3 (STAT3) activation and AMP-activated protein kinase (AMPK) deactivation, were induced by leptin, palm^11^-PrRP31, and their combination.

**TABLE 3:** Summary of metabolic and morphometric parameters in rodent models of leptin deficient or leptin signaling disturbances and impact of treatment with palm-PrRP31 or palm^11^-PrRP31.

Model	Characterization	BW change	Cumulative food intake	Liver weight	Glucose	Insulin	Leptin	TAG	CHOL	FFA
*ob/ob*	*ob/ob* vs WT	↑ 82%	NS	↑	NS	↑	NT	NS	↑	NS
MSG	MSG vs controls	↑^ns^ 7.5%	NT	NT	NS	↑	↑	NT	NT	NT
ZDF	Diabetic ZDF vs non-diabetic ZDF	↑ 10%	↑	↑	↑	NS	↑	↑	↑	NS
SHROB	SHROB vs SHR	↑ 38%	NS	↑	NS	↑	↑	↑	NS	↓
*ob/ob*	palm^11^-PrRP31	↓^ns^ 4%	NS	NS	NS	NS	NT	NS	↓	NS
MSG	palm-PrRP31	↓^ns^ 5.6%	↓	NT	NS	NS	NS	NT	NT	NT
ZDF	palm-PrRP31	↓^ns^ 2%	↓	NS	NS	NS	NS	NS	↓	NS
SHROB	palm^11^-PrRP31	↓^ns^ 1.5%	↓	NS	NS	↓	NS	NS	NS	↑

Statistical analysis was performed by unpaired *t*-test, significance is shown as increase (↑) or decrease (↓) vs their age-matched controls or treatment vs saline treated group. Cummulative food intake and body weight (BW) change measured at the end of experiment. Cholesterol (CHOL), free fatty acid (FFA) and triacylglycerides (TAG) measured from the plasma. Koletsky rats or spontaneously hypertensive obese rats (SHROB), monosodiumm glutamate (MSG), Non-significant (NS), not-tested (NT), Zucker diabetic fa/fa rats (ZDF), (Maletínská et al., 2015; Holubova et al., 2016; Pražienková et al., 2017; Holubová et al., 2018; Mikulaskova et al., 2018; Čermáková et al., 2019; Korinkova et al., 2020).

Our study (Korinkova et al., 2020) clearly showed that palm^11^-PrRP31 and leptin synergistically lowered BW (Figure 3) and increased body temperature in older *ob/ob* mice with established morbid obesity. However, the effect of the combination of both drugs on liver weight was only seen in younger *ob/ob* mice. We can conclude that palm^11^-PrRP31 might be a potential anti-obesity drug in the case of a functional leptin system.

MSG mice are a widely used rodent model of obesity and prediabetes. This model is induced by subcutaneous injections of monosodium glutamate (MSG) administered to newborns, resulting in specific lesions in the Arc of the hypothalamus (Takasaki 1978), leading to prediabetes with mild hyperglycemia, hyperinsulinemia and hyperleptinemia (Cameron, Poon, and Smith 1976; Matysková et al., 2008). The obesity of these animals is characterized by increased adiposity (Djazayery, Miller, and Stock 1979) because of a lower metabolic rate rather than elevated food intake (Maletínská et al., 2006). We tested whether treatment with palm-PrRP31 influenced the metabolic parameters of the MSG model at 6 months of age when the total adipose tissue weight and plasma level of leptin were significantly higher.

Two weeks of SC treatment with palm-PrRP31 did not significantly change BW or plasma leptin levels, while the white adipose tissue weight tended to decrease after treatment (Špolcová et al., 2015). While MSG mice were normoglycemic, plasma insulin levels were significantly higher in the MSG mice than in age-matched controls (Table 3). The cumulative food intake was significantly decreased after treatment with palm-PrRP31, but the fasting glucose and insulin levels did not differ from those in the saline-treated controls (Table 3). An intraperitoneal glucose tolerance test (IPGTT) showed that only the final glucose level was significantly lower in MSG mice treated with palm-PrRP31 than in MSG mice treated with saline (Špolcová et al., 2015). Moreover, palm-PrRP31 appeared to exert a central anorexigenic effect, resulting in increased phosphorylation of the insulin cascade kinases phosphoinositide-dependent protein kinase 1 (PDK1), protein kinase B (Akt) and glycogen synthase kinase-3β (GSK-3β).

We can conclude that palm-PrRP31 affects metabolic parameters connected with prediabetes in the periphery of MSG mice and insulin signaling in the hippocampus without an effect on BW.

Zucker diabetic rats, which are a model of impaired leptin receptor signaling (Fellmann et al., 2013), are frequently used for studying the potential of antiobesity and antidiabetic peptidic drugs (Andreassen et al., 2014; Skarbaliene et al., 2015). Thus, we used this model to evaluate the chronic antidiabetic potency of palm-PrRP31 and the involvement of the leptin signaling pathway in these effects.

As evident from the definition of this model, ZDF rats were slightly overweight and highly hyperglycemic compared to controls (Holubova et al., 2016). Diabetic ZDF rats had significantly increased cumulative food intake and hyperglycemia and exhibited markedly lowered glucose tolerance during the OGTT in comparison with controls. Hyperlipidemia was also found in diabetic ZDF rats *via* significantly increased plasma cholesterol and TAG in comparison with controls (Table 3) (Holubova et al., 2016). In this model, 2 weeks of treatment with palm-PrRP31 did not affect BW but had a tendency to improve tolerance to glucose but did not affect fasting glucose. However, the treatment lowered food intake and significantly decreased plasma cholesterol and nonsignificantly decreased plasma free fatty acids, triglycerides, leptin and insulin levels (Table 3).

This study clearly demonstrated that despite the food intake-lowering effect, palm-PrRP31 failed to decrease BW or improve glucose tolerance in this model, probably again due to a lack of functional leptin receptors and therefore the impossibility of an interaction of leptin and PrRP systems in the brains of ZDF rats.

The Koletsky rat strain of genetically obese hypertensive rats develops obesity, hyperinsulinemia, hyperlipidemia and spontaneous hypertension, which are the main symptoms of MetS (Koletsky 1973; Xu et al., 2008). These rats showed elevated fasting insulin levels compared to lean spontaneously hypertensive rats (SHRs), which were used as a control. OGTTs also demonstrated glucose intolerance; however, the rats were not diabetic, as previously reported (Friedman et al., 1997). Therefore, we tested parameters and insulin signaling in SHROB rats and their SHR controls.

**FIGURE 4:**
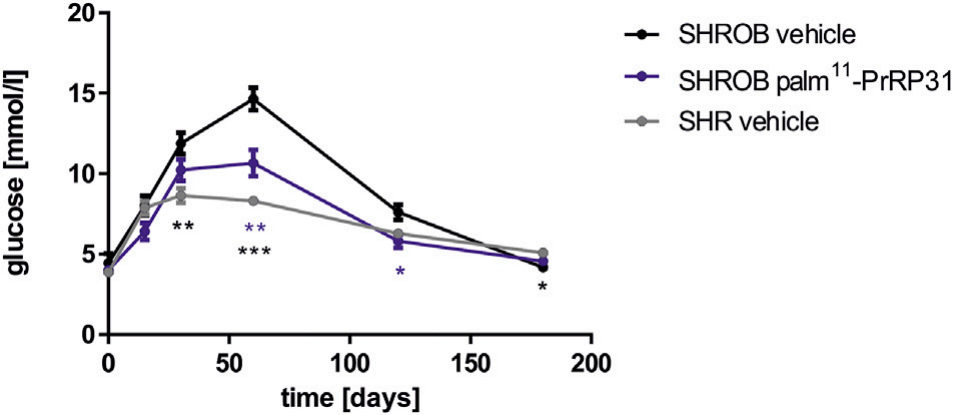
Effect of chronic treatment with palm^11^-PrRP31 on glucose tolerance response in SHROB rats. Rats were treated with palm^11^-PrRP31 for 3 weeks (SHROB palm^11^-PrRP31 group). Oral glucose tolerance test (OGTT) was performed after overnight fasting at the end of experiment. Results are shown as a glucose profile modified from (Mikulaskova et al., 2018). Data are presented as means ± S.E.M. Statistical analysis was performed by Two-way ANOVA with Tukey *post hoc* test, significance is *p*<0.05, **<0.01, ***<0.001 vs. the vehicle treated control SHR group.

As expected, SHROB rats were obese and had higher leptin, cholesterol and triglyceride levels than SHR controls (Table 3). The level of insulin was significantly higher than that in controls, while both genotypes were normoglycemic (Table 3). SHROB rats showed significantly higher liver weights than SHRs, but kidney and heart weights did not show differences between genotypes in which both were hypertensive (Mikulaskova et al., 2018). The mRNA expression levels of several genes related to lipogenesis in the liver or in adipose tissue were significantly higher in SHROB rats than in SHR controls. Stearoyl-CoA desaturase 1 (Scd-1) contributes to the development of obesity and is suppressed by functional leptin (Biddinger et al., 2006); thus, in this strain with a mutation in the leptin receptor, subcutaneous adipose tissue (SCAT) mRNA expression of Scd-1 was significantly higher in SHROB rats than in SHR controls (Mikulaskova et al., 2018). Treatment with palm^11^-PrRP31 for 3 weeks lowered food intake in both genotypes; however, an effect on BW was seen only in the SHR group with intact leptin signaling. While fasting plasma glucose levels were not affected by treatment in either genotype, based on OGTT results, palm^11^-PrRP31 administration significantly improved tolerance to glucose (Figure 4) in both groups and improved insulin signaling in the hypothalamus (Mikulaskova et al., 2018). The treatment did not have any effect on hypertension in either strain.

The most important result of this study was the marked improvement in glucose tolerance after palm^11^-PrRP treatment in both genotypes, while fasting normoglycemia was not altered. This improvement in glucose tolerance was accompanied by a significant decrease in plasma insulin levels and improved central insulin signaling in SHROB rats. The results also suggested that intact leptin signaling is needed for the BW-lowering effect.

## Conclusion

This review summarizes our results with a novel potential anorexigenic drug, palmitoylated PrRP, showing its effects on several parameters characterizing obesity or T2DM in different rodent models. Each of these models has specific features and might help us to analyze the particular effects of anorexigenic palm-PrRP analogs and to depict their mechanism of action as potential antiobesity and antidiabetic compounds.

DIO rodent models developed severe obesity, prediabetes or diabetes, resulting in BW gain that was mediated by an increase in body fat and liver weight; in addition, these models showed an increased level of leptin, with disturbed metabolic parameters and increased lipogenesis in adipose tissue. Palm-PrRP31 and palm^11^-PrRP31 seems to reverse the effects of a HF diet. A decrease in food intake resulted in attenuated fat storage and body and liver weight, accompanied by a decrease in leptin levels. Furthermore, palmitoylated analogs of PrRP affected lipid metabolism in adipose tissue and the liver by suppressing lipid synthesis and increasing lipid degradation. Moreover, increased mRNA expression of UCP-1 in BAT points to increased energy expenditure. A very interesting result was also demonstrated in the study after the treatment was discontinued: no yo-yo effect was observed after palm^11^-PrRP31 treatment termination.

The rodent models of leptin deficiency or disturbances in leptin signaling mentioned in this review developed obesity or morbid obesity, but treatment with palm-PrRP31 or palm^11^-PrRP31 did not significantly decrease BW or related metabolic parameters. On the other hand, treatment of *ob/ob* mice with a combination of leptin and palm^11^-PrRP31 synergistically decreased BW. This synergistic effect was also confirmed by a lower liver weight and body fat and increased body temperature. In two rat strains with nonfunctional leptin signaling, ZDF diabetic rats and Koletsky rats, monotherapy with palm^11^-PrRP31 or palm-PrRP did not have an antiobesity effect, but there were significant glucose-lowering effects. These results suggest that to achieve the full anti-obesity effects of PrRP, intact leptin signaling is needed, but the effect on glucose tolerance could be independent of leptin signaling. The central effect of both palmitoylated PrRP analogs was demonstrated by increased leptin and insulin signaling in the brain.

**FIGURE 5:**
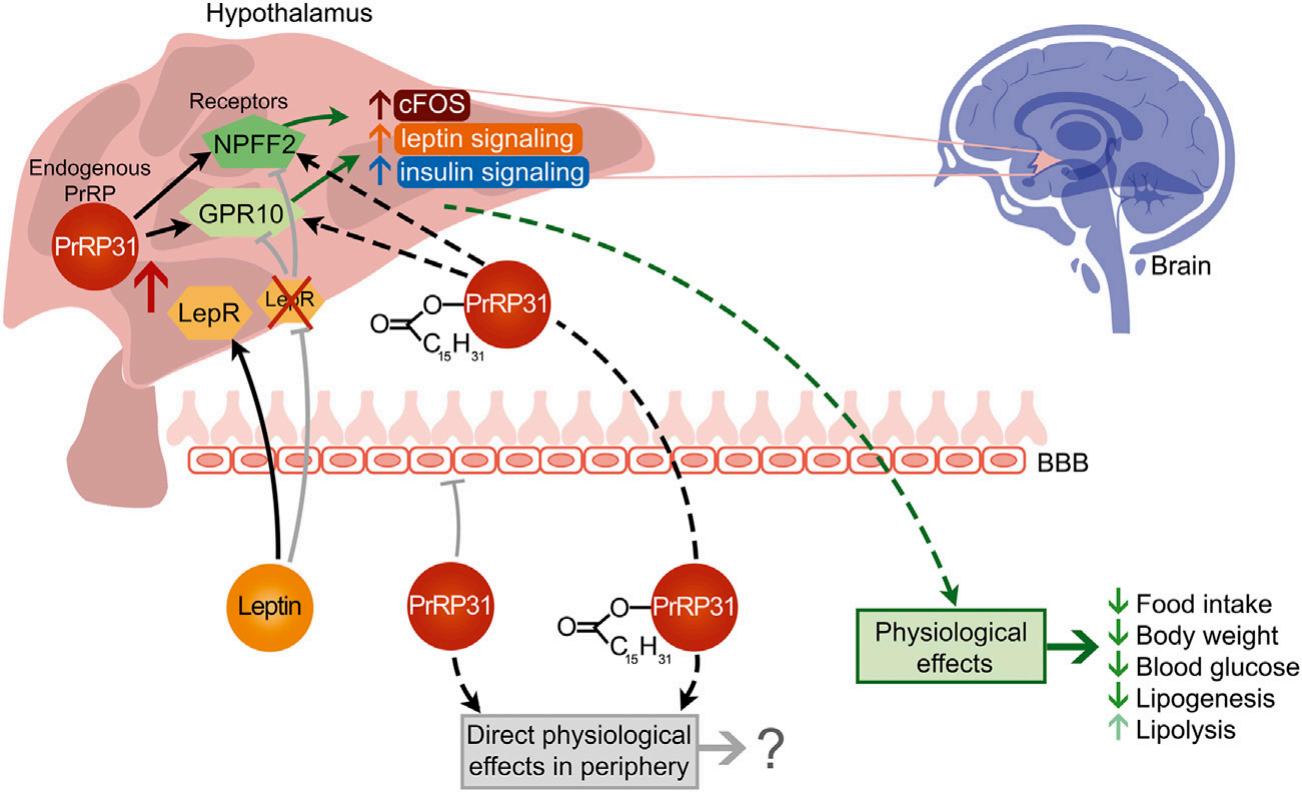
Proposed peripheral and central action of natural PrRP31 and its palmitoylated analog.

Overall, based on the results described in this review and in our other studies, the effects of palmitoylated PrRP analogs are summarized in Figure 5. It is evident that natural PrRP is not able to act centrally after peripheral administration and thus affects BW and related metabolic parameters. On the other hand, palmitoylated PrRP stimulates anorexigenic pathways in the hypothalamus. However, our results clearly suggest that the central effects of peripherally applied palm-PrRP on food intake and BW are possible only in the presence of intact leptin signaling. Despite this, palmitoylated PrRP has the potential to be an attractive candidate for obesity therapy.

## Glossary

*Acaca*: acetyl-CoA carboxylase
Akt: protein kinase B
AMPK: AMP-activated protein kinase
Arc: nucleus arcuatus
BAT: brown adipose tissue
BBB: blood brain barrier
BW: body weight
CART: cocaine- and amphetamine-regulated transcript
CCK: cholecystokinin
CHOL: cholesterol
DIO: diet induced obesity
DMN: dorsomedial nucleus
ERK: extracellular signal regulated kinase
*Fasn*: fatty-acid synthase
FFA: free fatty acid
GLP-1: glucagon-like peptide 1
GPR10: G-protein coupled receptor
GSK-3β: glycogen synthase kinase-3β
HF: high fat
IP: intraperitoneal
IPGTT: intraperitoneal glucose tolerance test
*Irs*: insulin receptor substrate
JNK: c-Jun N-terminal kinase
LF: low fat
MCH: melanin-concentrating hormone
MetS: metabolic syndrome
Myr-PrRP31: myristoylated PrRP
NPFF: neuropeptide FF
NPY: neuropeptide Y
NTS: nucleus tractus solitarii
OGTT: oral glucose tolerance test
Palm^**11**^-PrRP31: palmitoylated PrRP at position 11
Palm-PrRP31: palmitoylated PrRP
PDK1: phosphoinositide-dependent protein kinase 1
PI3K: phosphatidylinositol-3-kinase
PrRP: prolactin releasing peptide
PVN: paraventricular nuclei
SC: subcutaneous
SCAT: subcutaneous adipose tissue
*Scd-1*: stearoyl-CoA desaturase-1
*Srebp*: sterol regulatory element-binding protein
STAT3: signal transducer and activator of transcription-3
T2DM: type 2 diabetes mellitus
TAG: triacylglycerides
*UCP-1*: uncoupling protein 1
WT: wild type
α-MSH: α-melanocyte-stimulating hormone
